# Supplementary material for: Tissue-Specific Effects of the DNA Helicase FANCJ/BRIP1/BACH1 on Repeat Expansion in a Mouse Model of the Fragile X-Related Disorders
Source: Int J Mol Sci. 2025 Mar 15;26(6):2655. doi: 10.3390/ijms26062655 (PMC11942155; doi:10.3390/ijms26062655)
Supplement: Supplementary file 1 [file ijms-26-02655-s001.zip › ijms-3435063-supplementary.pdf]

Tissue-Specific Effects of the DNA helicase FANCJ/BRIP1/BACH1 on Repeat Expansion in a Mouse Model of the Fragile X-Related Disorders

Diego Antonio Jimenez [†], Alexandra Walker, Karen Usdin [*], and Xiaonan Zhao [*]

Section on Gene Structure and Disease, Laboratory of Cell and Molecular Biology, National Institute of Diabetes and Digestive and Kidney Diseases, National Institutes of Health, Bethesda, MD 20892, USA; diego.jimenez@pennmedicine.upenn.edu (D.A.J.); alexandra.walker@nih.gov (A.W.)

* Correspondence: karenu@nih.gov (K.U.); xiaonan.zhao@nih.gov (X.Z.); Tel.: +1-301-496-2189 (K.U.); +1-301-451-6322 (X.Z.)

† Current address: Department of Neuroscience, Perelman School of Medicine, University of Pennsylvania, Philadelphia, PA 19104, USA

Keywords: *FMR1* gene; somatic instability; DNA helicase; FANCJ; repeat expansion diseases; genetic modifier

---

Supplementary Figures

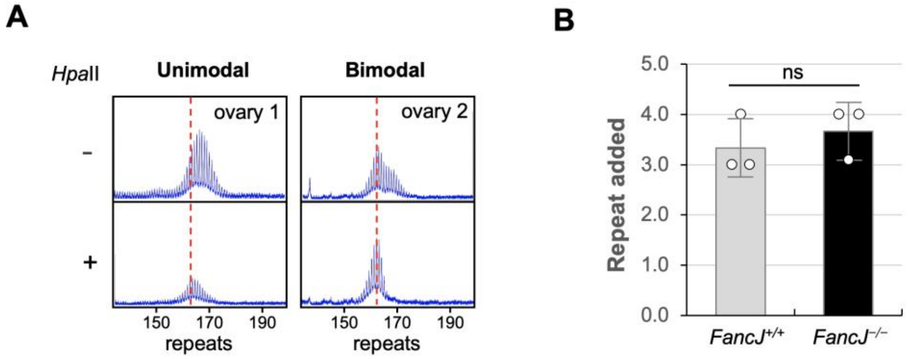

Figure S1. The effect of loss of FANCJ on repeat expansion in ovary of FXD mice at 6 months of age. **(A)** Representative repeat PCR profiles from ovaries with either a unimodal (left) or bimodal (right) pattern resulting from variations in X chromosome inactivation (XCI). Genomic DNA from mouse ovaries were treated without (-) or with (+) *Hpa*II before PCR. The dashed lines represent the sizes of the original inherited alleles as ascertained from the tail DNA taken at 3 weeks. The ovary having a unimodal profile in the absence of *Hpa*II shows only a relatively small amount of residual methylated allele after *Hpa*II predigestion. This is consistent with most of the FXD allele being on the active X chromosome. In contrast, the ovary with the bimodal PCR profile without *Hpa*II predigestion has a larger amount of residual methylated allele after *Hpa*II digestion. The bimodal profile makes it difficult to accurately determine any change in repeat number on the active X chromosome. Thus, for the sake of simplicity, only alleles with a unimodal PCR profile were used to ascertain the number of repeats added. **(B)** Comparison of the repeat added number in the ovary of 6-month-old *FancJ*$^{+/+}$ and *FancJ*$^{-/-}$ FXD mice with an average of 164 repeats in the original allele. The data represents the average of 3 *FancJ*$^{+/+}$ and 3 *FancJ*$^{-/-}$ mice with 160–167 repeats and unimodal PCR profiles. The error bars indicate the standard deviations of the mean. Each dot represents one animal. The number of repeats added was compared using two-tailed unpaired t-test. ns, not significant.

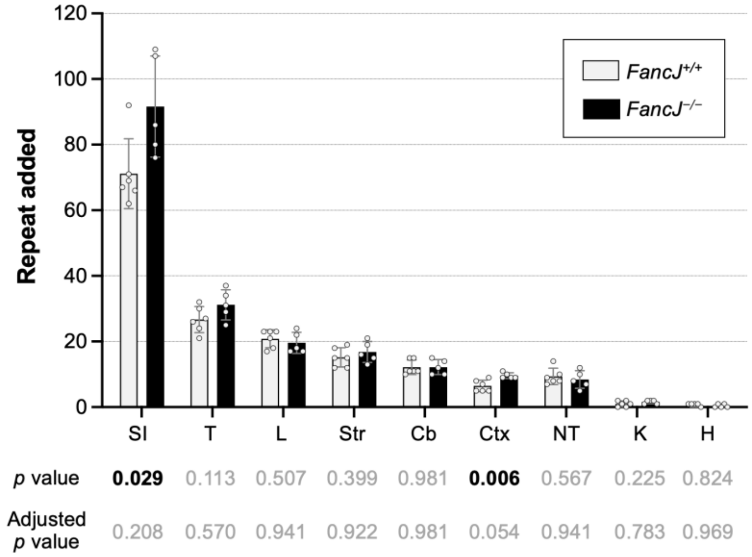

Figure S2. The effect of loss of FANCJ on repeat expansion in different tissues of 12-month-old FXD mice. Comparison of the number of repeats added in the indicated organs of 12-month-old *FancJ*$^{+/+}$ and *FancJ*$^{-/-}$ FXD mice with an average of 166 repeats in the original allele. The data represents the average of 6 *FancJ*$^{+/+}$ and 5 *FancJ*$^{-/-}$ mice in the repeat range of 160–170. The error bars indicate the standard deviations of the mean. Each dot represents one animal. In each organ, the repeat added for different genotypes were compared using unpaired t-test with Holm-Šídák's multiple comparisons correction. The *p*-values and adjusted *p*-values are listed in the table below. SI, small intestine; T, testes; L, liver; Str, striatum; Cb, cerebellum; Ctx, cortex; NT, tail; K, kidney; H, heart.
